# Supplementary figures and images for: Concurrent exercise training induces additional benefits to hydrochlorothiazide: Evidence for an improvement of autonomic control and oxidative stress in a model of hypertension and postmenopause
Source: PLoS One. 2023 Aug 7;18(8):e0289715. doi: 10.1371/journal.pone.0289715 (PMC10406179; doi:10.1371/journal.pone.0289715)

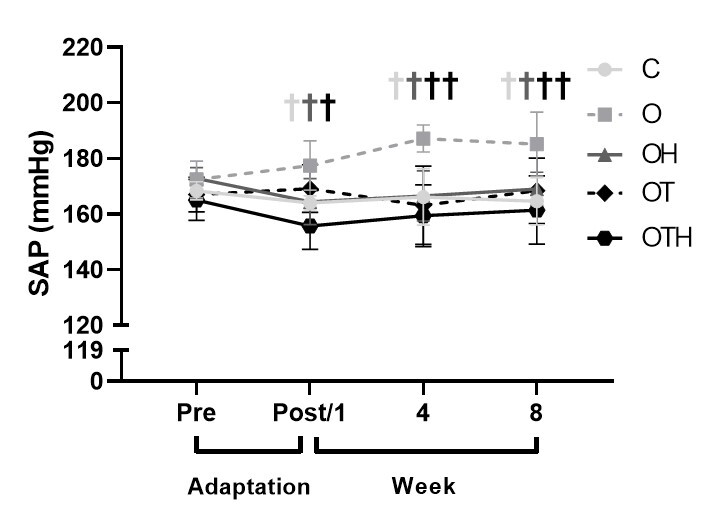

Supplement: S1 Fig — (DOCX) [file pone.0289715.s001.docx]
