# Supplementary material for: Concurrent exercise training induces additional benefits to hydrochlorothiazide: Evidence for an improvement of autonomic control and oxidative stress in a model of hypertension and postmenopause
Source: PLoS One. 2023 Aug 7;18(8):e0289715. doi: 10.1371/journal.pone.0289715 (PMC10406179; doi:10.1371/journal.pone.0289715)
Supplement: S1 Table — (DOCX) [file pone.0289715.s002.docx]

| **S1 Table. Correlation analysis involving all studied groups.** | | |
| --- | --- | --- |
| **Var-SAP** | R | p |
| SAP | 0.58 | <0.001 |
| RPP | 0.64 | <0.001 |
| LF-SAP | 0.89 | <0.001 |
| LF/HF | 0.53 | 0.002 |
| IL-10/TNF-α | -0.46 | 0.006 |
| **LF-SAP** |  |  |
| SAP | 0.43 | 0.007 |
| RPP | 0.60 | <0.001 |
| LF/HF | 0.52 | 0.002 |
| IL-10/TNF-α | -0.56 | <0.001 |
| Data were analyzed using Pearson correlation. 7-8 rats/group. Var-SAP, variance of systolic arterial pressure; SAP, systolic arterial pressure; RPP, rate-pressure product; LF, low frequency band; HF, high frequency band; IL-10, interleukin 10; TNF-α, tumor necrosis factor alpha; LF-SAP, low frequency band of systolic arterial pressure. | | |
