# Supplementary material for: Concurrent exercise training induces additional benefits to hydrochlorothiazide: Evidence for an improvement of autonomic control and oxidative stress in a model of hypertension and postmenopause
Source: PLoS One. 2023 Aug 7;18(8):e0289715. doi: 10.1371/journal.pone.0289715 (PMC10406179; doi:10.1371/journal.pone.0289715)
Supplement: S1 File — (DOCX) [file pone.0289715.s003.docx]

# **Supporting Information Files**

## **Detailed methods**

### **Ovariectomy**

The rats were ovariectomized at 90 days of age. Only the control (C) group was not subjected to ovariectomy. The surgery was performed as described previously [1] with slight modifications. Meloxicam (1 mg/kg) was administered subcutaneously 30 minutes before surgery for analgesic effect. After that, an *ip* doses of ketamine (80 mg/kg) and xylazine (12 mg/kg) were injected for anesthetize. Rats are placed in dorsal decubitus for a small incision (1 cm) in parallel with the body line in the skin and muscles in the lower third in the abdominal region. After location of ovaries, the ligation of the oviducts was performed, including the blood vessels. The oviducts were sectioned and both ovaries removed. The muscle wall and skin were then sutured with nylon and silk thread, respectively. An *im* dose of penicillin (10000 U/kg) was administered after surgery. In addition, doses of meloxicam (1 mg/kg) were administered every 24 hour and tramadol (20 mg/kg) every 8 hours, for 3 days, for analgesic effects.

### **Tail-cuff plethysmography**

All groups underwent an adaptation to the equipment for 3 days. After that, AP measurements were performed before the 7 days of medication adaptation, post medication adaptation/day 1 of the intervention period, fourth and eighth week of the intervention period.

The rat was positioned on a heated platform and a steel container adjusted to its size was used to keep it in a condition of minimal movement. A cuff with a pneumatic pulse sensor was positioned near the base of the rat's tail to occlude blood flow during measurements and the tail was gently fixed with tapes to contain the movement. Approximately 10-15 “warm up” measurements were performed, until a good acquisition of the tail pulse signal was achieved, and then the measurements that would be considered valid for the study were initiated.

Were performed 20 consecutive and uninterrupted measurements, with an interval of 15 seconds between each measurement. Systolic AP (SAP) values ​​were recorded (model BP-2000 Blood Pressure Analysis System, Visitech Systems, Inc, North Carolina, USA) at the same time of day with all groups studied to avoid circadian cycle influences. The procedure was performed with up to 2 rats at the same time. Platform temperature (36°C) and other settings were adjusted according to the equipment manufacturer's recommendations. Resting SAP was considered to be the mean of at least 10 valid measurements. A new attempt was performed on another day if the minimum amount of valid measurements determined for the study was not obtained.

### **Concurrent exercise training protocol**

Rats underwent to the first tests in the second week following ovariectomy. Maximal running test consisted of an initial speed 0.3 km/h without inclination of the treadmill. After every 3 minutes, the speed was increased in 0.3 km/h until voluntary exhaustion of the rats [2].

The maximal load test was performed one day after treadmill test. Initially, the rats climbing 2 times without external overload. After that, a load of 15% of the body weight was externally added to each subsequent climb. All climbs were performed after 2-min resting period. The test ended when the rat was unable to climb the ladder to the top [3]. No aversive resources were used as a stimulus to maintain performance for running and climbing. However, incentives were given with a touch, with the hand, on the tail base, when necessary.

The test was performed at the beginning of the experiment (post drug adaptation), as well as the fourth and eighth weeks of the exercise training (intervention period). For the treadmill and ladder exercise prescription, the maximal speed (km/h) and load (grams) reached by each rat during the respective tests was considered and was adjusted weekly, according to the sessions. At fourth week, new tests were carried out and the aerobic and resistance exercises training were adjusted considering the recent performance of the rats.

**Aerobic exercise training:** Aerobic exercise training was performed predominantly at moderate intensity (40-60% of the maximal running speed obtained in the first test). In the initial 12 sessions the rats trained for 30 minutes, while from the 13 to 24 sessions the training lasted 30-40 minutes.

**Resistance exercise training:** Resistance exercise training was performed immediately after aerobic exercise and predominantly at moderate intensity (40-60% of the maximal load obtained in the first test). The protocol consisted of 15 (1-12^th^ sessions) to 15-20 climbs (13-24^th^ sessions) and 1-min time interval between climbs, for 3 days a week.

The order of exercises (first aerobic followed by the resistance exercise) is because we choose to give preference to aerobic exercise as it is currently the exercise model with the most well-established results in the AP control and treatment of AH.

### **Inflammation assessment in cardiac tissue**

For inflammation analyses, the preparation of cardiac tissue was initiated by cutting the tissue into pieces and adding 400μL of 100 mM Tris buffer with 10 mM EDTA and pH 7.5, 4μL of protease inhibitor, and 40μL of 1% Triton X-100. The tissue was then homogenized for 30 seconds using an Ultra-Turrax homogenizer, centrifuged for 30 minutes at 12.000 rpm in a refrigerated centrifuge (between 0 and 4°C), and the supernatant was frozen at -80°C for further assays.

The measurements of TNF-α, IL-6, and IL-10 in the heart were performed in microplates (96 wells) sensitized with the antibody for the protein of interest, which was immobilized on the plate wells by an immune absorbent substrate. Prior to that, a blocking step was performed to prevent nonspecific binding, followed by incubation with the experimental samples containing the protein (antigen) to be measured. This was followed by incubation with an enzyme-linked antibody and subsequent reaction with a chromogen. A standard curve was also included on the plate, which was used to calculate the amount of protein per well. Absorbance was measured using an ELISA reader.

Specific kits for rats were used: TNF-α, IL-6, and IL-10 (R&D Systems). The assay for measurement used specific standard curve concentrations for each substance.

### **Oxidative stress assessment in cardiac tissue**

**Lipoperoxidation measurement by Thiobarbituric Acid Reactive Substances (TBARS)**

For the thiobarbituric acid reactive substances (TBARS) assay, trichloroacetic acid (10%, w/v) was added to the homogenate to precipitate proteins and to acidify the samples. This mixture was then centrifuged (3.000 rpm, 3 min), the protein-free sample was extracted, and thiobarbituric acid (0.67%, w/v) was added to the reaction medium. The tubes were placed in a water bath (100 °C) for 15 min. Absorbance was measured at 535 nm using a spectrophotometer. The results are expressed as micromoles (per milligram of protein) [4].

**Protein oxidation**

This method uses the reaction of protein carbonyl groups with 2,4-dinitrophenylhydrazine (DNPH) to form a 2,4-dinitrophenylhydrazone, which can be measured spectrophotometrically at 360 nm as previously described [5].

**NADPH oxidase**

Nicotinamide adenine dinucleotide phosphate (NADPH) oxidase activity was evaluated by superoxide production determined by plate reading. Phosphate buffer 50mM pH 7.4 with 2mM EDTA and 150mM sucrose, 1.3mM NADPH and 10 µL of sample were used. Superoxide production was expressed in μmoles/mg of protein [6]. The reading was performed at 340nm.

**Hydrogen peroxide**

Hydrogen peroxide (H_2_O_2_) concentration was assessed based on the horseradish peroxidase- (HRPO) mediated oxidation of phenol red by H_2_O_2_ [7].

**Antioxidant enzymes**

Superoxide dismutase (SOD) activity was measured spectrophotometrically in heart homogenates by rate inhibition of pyrogallol autooxidation at 420 nm [8]. Catalase (CAT) concentration was measured by monitoring the decrease in H_2_O_2_ concentration at 240 nm [9]. Glutathione peroxidase (GPx) activity was determined by monitoring NADPH oxidation spectrophotometrically at 340 nm [10].

**Ferric Reducing Antioxidant Power (FRAP)**

The determination of non-enzymatic antioxidant activity measured by the ferric reducing antioxidant power (FRAP) is based on the production of Fe2+ ion (ferrous form) from the reduction of Fe3+ ion (ferric form) present in 2,4,6-tripyridyl-s-triazine (TPTZ) complex. Thus, the change in absorbance is directly related to the power of the total reduction of antioxidant electron donation present in the reaction. The technique was performed in a microplate, in which 290μL of FRAP reagent (sodium acetate and acetic acid buffer, pH 3.6; TPTZ 10mM; ferric chloride hexahydrate, 20mM), 10 μL of a standard solution of ferrous sulfate heptahydrate and 10 μL of sample. The microplates were measured spectrophotometrically at 593 nm [11]. The values were expressed in mM of Fe (II).

**Nitrite**

Nitrite levels were measured by reacting samples with Griess' reagent in microplates (96 wells) in an ELISA reader device. On the plate, 50 µl of Griess reagent was added in aliquots of 50 µl of sample at room temperature [12]. Total tissue nitrite was estimated from a standard absorbance curve at 545 nm.

# **Supplemental references**

1. Irigoyen MC, Paulini J, Flores LJF, Flues K, Bertagnolli M, Moreira ED, et al. Exercise training improves baroreflex sensitivity associated with oxidative stress reduction in ovariectomized rats. Hypertension. 2005;46(Part 2):998–1003.

2. Rodrigues B, Figueroa DM, Mostarda CT, Heeren M V., Irigoyen MC, De Angelis K. Maximal exercise test is a useful method for physical capacity and oxygen consumption determination in streptozotocin-diabetic rats. Cardiovasc Diabetol. 2007 Dec 13;6:38.

3. Sanches IC, Conti FF, Sartori M, Irigoyen MC, De Angelis K. Standardization of resistance exercise training: effects in diabetic ovariectomized rats. Int J Sports Med. 2014;35(4):323–9.

4. Ohkawa H, Ohishi N, Yagi K. Assay for lipid peroxides in animal tissues by thiobarbituric acid reaction. Anal Biochem. 1979;95(2):351–8.

5. Reznick AZ, Packer L. Oxidative damage to proteins: spectrophotometric method for carbonyl assay. Methods Enzymol. 1994 Jan 1;233:357.

6. Wei Y, Sowers JR, Nistala R, Gong H, Uptergrove GME, Clark SE, et al. Angiotensin II-induced NADPH oxidase activation impairs insulin signaling in skeletal muscle cells. J Biol Chem. 2006 Nov 17;281(46):35137–46.

7. Pick E, Keisari Y. A simple colorimetric method for the measurement of hydrogen peroxide produced by cells in culture. J Immunol Methods. 1980;38(1–2):161–70.

8. Marklund SL, Oreland L, Perdahl E, Winblad B. Superoxide dismutase activity in brains from chronic alcoholics. Drug Alcohol Depend. 1983;12:209–2015.

9. Boveris A, Chance B. The mitochondrial generation of hydrogen peroxide. General properties and effect of hyperbaric oxygen. Biochem J. 1973;134(3):716.

10. Flohé L, Günzler WA. Assays of glutathione peroxidase. Methods Enzym. 1984;105:114–20.

11. Benzie IF, Strain JJ. Ferric reducing/antioxidant power assay: direct measure of total antioxidant activity of biological fluids and modified version for simultaneous measurement of total antioxidant power and ascorbic acid concentration. Methods Enzymol. 1999;299:15–27.

12. Granger DL, Anstey NM, Miller WC, Weinberg JB. Measuring nitric oxide production in human clinical studies. Methods Enzymol. 1999;301:49–61.
