# Supplementary material for: Concurrent exercise training induces additional benefits to hydrochlorothiazide: Evidence for an improvement of autonomic control and oxidative stress in a model of hypertension and postmenopause
Source: PLoS One. 2023 Aug 7;18(8):e0289715. doi: 10.1371/journal.pone.0289715 (PMC10406179; doi:10.1371/journal.pone.0289715)
Supplement: Attachment — Submitted filename: Response to Reviewers.docx [file pone.0289715.s004.docx]

*Manuscript: PONE-D-23-10192R1*

*The authors would like to thank the reviewers for their valuable comments. We carefully considered all of the suggestions and accomplished with all of the requests within the aim and possibilities of the present study.* ***Responses to each question are provided below, and changes made in the manuscript are highlighted****.*

**Reviewer #1:**

**In the current study the authors investigated the effect of exercise in combination with HCTZ in ovariectomized SHRs. The animal experiment mimics a relevant clinical situation in which postmenopausal females are at higher risk of arterial hypertension and subsequent hypertension-dependent end-organ damage.**

**The strength of the study is the analysis of the effect of exercise on autonomic cardiovascular control. It is important to show that this exercise effect is not only stable in co-treatment with HCTZ but also slightly more improved in the combination. As expected this goes along with reduced inflammation in the heart. The weakness of the current study is the lack of any endo-organ functional data such as heart function (LVEF etc) or renal function (like proteinuria etc.). It is also important to make clearer that the BP was not reduced in exercise performing rats but the age-dependent further increase was attenuated. Thus, exercise contributes to the subsequent further development of hypertension and related disease but is not sufficient to reduce blood pressure. In the clinical meta-analysis cited in the study (Ref. 15) all exercise performing patients had a reduced body weight and were over-weight. Therefore, unlike the well conducted animal study here, this reference is misleading and does also not share the common view of the literature in which a blood pressure reducing effect of SHRs is more critically discussed.**

*Thank you for initial considerations and for opportunity to discuss this manuscript. Our results showed that SAP was reduced in C, OH, OT and OTH groups in relation to O group during the protocol (1^st^ to 8^th^ week, S1 Figure). These data demonstrated that both therapies (OH, OT and OHT groups) were able to attenuate ovarian hormones deprivation-dependent further increase in SAP in hypertensive rats in the present study. This information was added in the discussion (4^th^ paragraph). As mentioned, in the present study we did not present data on end-organ functional data such as heart function (LVEF, etc.) or renal function (like proteinuria, etc.). However, considering the reviewer comment, we calculated the Rate Pressure Product (RPP) =*  *Systolic Arterial Pressure* *(SAP) * Heart Rate (HR), which is a measure of the stress put on the* [*cardiac muscle*](https://en.wikipedia.org/wiki/Cardiac_muscle) *and is a direct indication of the energy demand of the heart and thus a good measure of the energy consumption of the heart. This data demonstrated heart functional improvement in exercise performing rats (OT and OHT groups) compared to the ovariectomized group (O) (previously referred to as group OS) and the control group (C). Moreover, RPP was correlated with VAR-SAP, LF-SAP, baroreflex and IL10/TNF-α. These data reinforce the impact of association of therapies, as suggested by the reviewer, and were discussed in this version of the manuscript. Regarding the reviewer's comment about the meta-analysis cited in our study (Ref. 15), we have chosen to exclude the citation for better alignment with the literature discussed.*

**Main points:**

**1. N numbers of animals are slightly different per group. This suggests that individual animals were removed from further analysis. Please state in the study why this was done.**

*Thank you for your comment.* *In some analysis we have problems in AP direct acquisition or in heart tissue measurements (no more than 1 animal per group for each measurement). The statistical analysis of each evaluation used the number of animals that data were obtained. We added the following sentence in the 1^st^ paragraph of the 'Materials and Methods' section: "**The rat that did not complete a specific evaluation was excluded from the statistical analysis."*

**2 – An interesting point not further mentioned or discussed by the reviewers is the lack of effect of exercise on IL6 expression. Is this because exercise performing SHRs had slightly bigger hearts?**

*We expected lower levels of heart IL-6 in the trained groups. Unfortunately, this result was not observed in our study. In a very similar exercise training model (however, 5 times a week vs. 3 times a week in the present study), we observed reduced levels of IL-6 in the cardiac tissue of female hypertensive rats (Shimojo et al, (2018) – DOI: 10.3389/fphys.2018.01471). We believe that the slightly higher heart weight in the trained group is predominantly related to a possible physiological adaptation induced by exercise training. However, we were unable to determine whether the absence of reduction in IL-6 levels is due to the slightly higher weight of cardiac tissue in the trained groups or if another mechanism is involved in this result.*

**3 – Body weights and heart weights are reported. Unfortunately, the heart weight is properly wrong because a heart weight of 0.7 mg for an SHR is unlikely. The study would further benefit from a report of normalized heart weights to body weight (if possible also to tibia length which would be more accurate). This is really informative as there are no further reports about the heart function.**

*We thank the reviewer observation and suggestion. We apologize that the unit of measurement used for heart weight was incorrect. Therefore, we have replaced "mg" per "g". Furthermore, we had previously presented left ventricular weight and mistakenly labeled it as heart weight. In this revised version, we have now provided the corrected heart weight. Despite the tibia was not collected, as requested by the reviewer, we added the normalized heart weight to body weight in the Table 1.*

**4 – It remains unclear what the information about SOD in cardiac tissue should explain. Is this SOD2 or SOD1 or both and what about SOD in plasma.**

*In this paper we included analysis of enzymatic and non-enzymatic antioxidant defenses, as well as measurements of pro-oxidants and tissue damage to allow a global view of oxidative stress in heart tissue. Moreover, we are focusing on heart tissue (to evaluate end organ damage) and did not perform any evaluation in plasma. We chose to include the measurement of the activity of the 3 key antioxidant enzymes (superoxide dismutase, catalase, and glutathione peroxidase) in the heart tissue. The measurements in heart homogenates included activity of both, SOD1 and SOD2 isoforms. We agree with the reviewer that the data on superoxide dismutase activity alone is not easy to be interpreted. In fact, SOD activity was not different between O and the other studied groups. These means that SOD activity in O group was like the reduced values observed in OH and OT groups, but also not decreased as compared to C or OTH groups. The integrated interpretation of our data demonstrated that heart NADPH oxidase and enzyme marked involved in superoxide anion generation, which is scavenger by SOD, was increased in O group (vs. C) and statically reduced in trained groups. Moreover, CAT (reduced vs. O) and GPx activities were markedly increased in OTH group (vs. O group), as observed for SOD (OTH vs. OH and OT groups). Together, these data suggest that the association of therapies (OTH group) induced more pronounced effect in enzymic antioxidant defenses. This information was included in the discussion.*

**5 – A limitation of the study is the lack of inflammatory markers in plasma as hypertension affects blood vessels.**

*We agree with the reviewer that measuring inflammatory markers in plasma would provide important information about the effects of hypertension on blood vessels in our experiment. However, the main objective of our study was to demonstrate whether the combination of hydrochlorothiazide and concurrent exercise training could positively modulate neuroinflammatory markers specifically in cardiac tissue. The heart is one of the main target organs affected during the development and progression of arterial hypertension. We think that the inclusion of RPP as a parameter of heart function, considering the reviewer suggestion, reinforce the focus of our objectives and the implication of our findings.*

**Reviewer #2:**

**The paper entitled “Concurrent exercise training induces additional benefits to hydrochlorothiazide: evidence for improve on autonomic control and oxidative stress in hypertensive rats” is very interesting. So, I will give you the opportunity to revise some points before resubmitting to the journal.**

*Thank you for initial considerations and for opportunity to review our work. All changes were highlighted in the manuscript.*

**Title: “Concomitant exercise training induces additional benefits to hydrochlorothiazide: evidence to improve autonomic control and oxidative stress in hypertensive rats”. There is an important point to be noted in the title that was not addressed, which would be postmenopausal hypertensive rats, not just hypertensive rats. I believe that as this study is based on looking for additional effects of HCTZ training in postmenopausal hypertensive animals, this should be considered when writing the title.**
 *We agree with the reviewer's consideration. Therefore, we have changed the title of the article to* **“***Concurrent exercise training induces additional benefits to hydrochlorothiazide: evidence for improve on autonomic control and oxidative stress in a model of hypertension and postmenopause”.*

**Abstract
Objective: “Although hydrochlorothiazide (HCTZ) is commonly prescribed to treat high blood pressure (BP), its role in restoring BP regulatory mechanisms is debatable”. “We focused to evaluate whether physical training could contribute to a better modulation of neurohumoral mechanisms in postmenopausal rats treated with HCTZ”. This session you should be more objective and less introductory to your topic.**

**Another point, the term high arterial pressure (AP) is not the ideal way to address arterial hypertension, because of the differences in having high blood pressure and being hypertensive. I suggest you review this concept.**

*We thank the reviewer’s comments. We have rewritten the objective of our study in the abstract, following the proposed suggestions by the reviewer. We rewritten the topic to: “The objective of the present study was to evaluate whether the exercise training could contribute to a better modulation of the neurohumoral mechanisms linked to the pathophysiology of hypertension in postmenopausal hypertensive rats treated with hydrochlorothiazide (HCTZ)”. In addition, we have considered changing the term "high arterial pressure (AP)" throughout our document as we understand that this term, indeed, would not be appropriate to use as a synonym for hypertension.*

**Methods: “Spontaneously hypertensive rats (SHR) were distributed into sedentary (S) and ovariectomized groups: sedentary alone (OS), treated with HCTZ alone (OSH), trained alone (OT) or combined with HCTZ (OTH).” improve this sentence, as it is not well understood whether all animals are from the SHR model or the ovariectomized animals are normotensive, explain the number of animals and days of life. Given the name sedentary to the untrained SHR group, is not the correct word to say, because animals, even if not trained, can generate energy expenditure inside the cage, for example, so the most correct name, can only be the hypertensive group or hypertensive control/no intervention group. For the sedentary alone (OS) group, ovariectomized hypertensive patients seem to be more correct. What techniques were used to evaluate your objective? needs to be introduced to make more sense of the methods.**

*Thank you for observations and suggestions. We have rewritten the methods to clarify the points raised by the reviewer. Considering the reviewer suggestion, we have changed the names of the groups as suggested to facilitate identification and reading.*

*We have also modified the abbreviation of each group name according to the new nomenclature used. These modifications were also made throughout the manuscript.*

*As requested, we have added in the abstract the main methods used in the study.*

**Keywords: "cardiovascular prevention", prevention or treatment, your animals already have hypertension and are being treated with hydrochlorothiazide, the use of the word prevention generates verbal disagreement with your methodology.**

*We agree about the incorrect use of the word "cardiovascular prevention". We have reviewed and replaced the term to "secondary prevention".*

**Introduction
It needs to be redone, the mechanisms studied in the article were not addressed in the introduction, what is the importance and influence of arterial hypertension on inflammation and oxidative stress? What does this imply in the regulation of autonomic mechanisms? How can the drug hydrochlorothiazide and exercise act on the regulation pathways of these autonomic and vasoconstrictor control mechanisms of arterial hypertension?**

*Considering the reviewer's comments, we have changed the introduction and included additional evidence regarding the mentioned topics. In the introduction, we have also emphasized the scientifically demonstrated inconsistencies regarding the ability of hydrochlorothiazide to modulate key mechanisms involved in arterial hypertension. Similarly, we have highlighted the mechanisms that are positively modulated by exercise training.*

**The use of acronyms in your article is not following the standards of publication with high blood pressure. 1- Arterial hypertension (HTN) this designation is incorrect the abbreviation HTN refers to hypertension in general, the more correct abbreviation term would be Arterial Hypertension (AH), emphasizing the type of hypertension studied. 2- arterial pressure (AP) or high arterial pressure (AP), is using the same abbreviation for two different things, in addition, if designating arterial hypertension as high arterial pressure is incorrect, because a person or animal that has arterial pressure high does not necessarily have the cardiovascular comorbidity of arterial hypertension.**

*We thank the reviewer’s comments.*

*1. We have revised the manuscript and replace the use of the acronym “HTN” for “AH”.*

*2. We have revised the manuscript and replace the use of the terms “arterial pressure” or “high arterial pressure” for another appropriate and assertive term.*

**In the sentence "The hydrochlorothiazide (HCTZ), a thiazide diuretic, has been one of the initial classes recommended in monotherapy or combined with other antihypertensive drugs, being widely used in clinical practice", is this treatment recommended by whom? Does this information come from a guideline? What recommended dose? How many times a day can everyone take this medication? Improve the phrase and refer to it to provide scientific support.**

*Thank you for this important observation. We agree about the incorrect use of term “recommended”. We revised the phrase again and the sentence was improved. We insert the current ‘Brazilian Guidelines for Hypertension - 2020’* *as a reference for our current sentence.*

**In this sentence: "promoting significant clinical reduction around −12 and −6 mmHg for SAP and DAP, respectively (15)" the acronyms SAP and DAP were not described or mentioned earlier in the text, what do they mean?**

*Considering the comments made by Reviewer#1 regarding the sentence containing reference (15), we have decided to exclude the sentence from the manuscript. Furthermore, we have reviewed the manuscript and inserted the acronyms "SAP" and "DAP" appropriately when mentioned earlier in the text.*

**In the sentence: "Despite this, exercise training induced chronic AP reduction is associated with adaptations in key AP control mechanisms such as autonomic, humoral, inflammation, and oxidative stress (17–21)". Necessary to be rewritten, for better emphasis and importance to the exercise. How does this happen? Which mechanism? Why study the baroreflex? Why study TNF-alpha, interleukins 6 (IL-6) and 10 (IL-10), how exercise modulates the pathway in hypertension? What is the importance of exercise in the redox state, does it decrease the increase of anti and pró oxidative enzymes? How is exercise related to vasoconstrictor or vasorelaxant mechanisms? How will all this together with hydrochlorothiazide treatment be beneficial? These points should be considered to base the objectives of your article, as they are mechanisms already studied and of great importance in the modulation of exercise and arterial hypertension.**

*We have rewritten the introduction following the proposed suggestions by the reviewer. We added sentences highlighting some of the adaptation induced by exercise training on mechanisms related to the pathophysiology of hypertension.*

**Another point not addressed by the authors, what is the influence of hormone deprivation induced by ovariectomized hypertension?**

*We have added a sentence highlighting this important additional harmful effect induced by ovarian hormone deprivation in an experimental model of hypertension at the end of the 2nd paragraph of the introduction.*

**Methodology
In this sentence: "Female spontaneously hypertensive rats (SHR) (150-200g, 90 days old) were obtained from Nove de Julho University (UNINOVE) (Sao Paulo, Brazil) and randomly allocated into (n=7−8 rats each group) sedentary (S), ovariectomized sedentary (OS), ovariectomized sedentary treated with hydrochlorothiazide (OSH), ovariectomized trained (OT) and ovariectomized trained and treated with hydrochlorothiazide (OTH)." Suggestion already mentioned about the denomination sedentary to groups not trained in physical exercise.**

*We have changed as suggested by the reviewer. Changes were highlighted in the manuscript.*

**Why did the authors use young rats aged 90 days (~12-13 weeks old) instead of older animals, which are already considered models of hypertension and postmenopause? Fortepiane (2002) suggests that 18-month-old postmenopausal rats, but not ovariectomized rats, may be a suitable model for the study of postmenopausal hypertension. Furthermore, SHR rats reach a stable level of hypertension at around 17-19 weeks of age, so why use ~12-13-week rats? Where have animals of other ages been examined in previous studies? Ref: Lourdes A. Fortepiani et al, (2002) - Characterization of an Animal Model of Postmenopausal Hypertension in Spontaneously Hypertensive Rats – https://doi.org/10.1161/01.HYP.0000046924.94886. EF Hypertension. 2003;41:640–645.**

*Thank you for opportunity to clarify this topic. Ovariectomized rats are utilized in biological research as a model to mimic menopause in women. We (Brito-Monzani et al, (2018) – DOI: 10.1038/hr.2017.94; Sanches et al, (2018) – DOI: 10.1371/journal.pone.0202731; Shimojo et al, (2018) – DOI: 10.3389/fphys.2018.01471; Ferreira et al, (2020) – DOI: 10.1186/s13293-020-00290-y) and others authors (Felix et al, (2015) – DOI: 10.1152/japplphysiol.00483.2014; De Melo et al, (2016) – DOI: 10.3389/fphys.2016.00461) have studied different models of ovarian hormone deprivation. The performance of early ovarian hormone deprivation in adult rats is justified by the fact that aging is a confounding factor in the analysis. Data obtained from rats undergoing ovarian hormone deprivation later in life (i.e., at more advanced ages) do not allow us to accurately determine whether the findings are a result of ovarian hormone deprivation or the aging process itself. By performing ovariectomy in adult rats, we eliminate the age/aging factor, enabling us to identify the impacts caused specifically by ovarian hormone deprivation. It is important to highlight that at the time of the procedure (90 days), the rats are already in their reproductive age, which facilitates our investigative procedure.*

*Our plethysmography data presented in the supplementary material (see S2 Fig.) (with measurements conducted on rats at approximately 14-15 weeks of age) demonstrate that all evaluated groups, including the non-ovariectomized group (currently referred to as the hypertensive control group (C)), already exhibited hypertension, with mean systolic arterial pressure values of 168 ± 3, 172 ± 7, 172 ± 8, 167 ± 6, and 164 ± 7 mmHg for groups C (previously referred to as group S), O (previously referred to as group OS), OH (previously referred to as group OSH), OT, and OTH, respectively. In addition, a steady state was observed throughout treatment, as seen by the 3 systolic arterial pressure measurements taken afterwards.*

**"Pharmacological treatment was performed using hydrochlorothiazide (Sanofi Medley Farmacêutica, Campinas, SP, Brasil), an antihypertensive drug corresponding to thiazide diuretics class, at a dose of 30 mg/kg/day. According to our pilot study conducted previously, this dose during 1 wk showed sufficient to promote an AP reduction of approximately 10−12 mmHg in ovariectomized SHR", previously published scientific and methodological support is necessary, this dose or another has possibly already been used by other authors, who sought the purpose of treating arterial hypertension.**

*We thank the reviewer for the comment. We have added published scientific and methodological support that investigated the arterial pressure-lowering effect produced by different doses of hydrochlorothiazide in hypertension.*

**"The hydrochlorothiazide tablet was macerated, diluted in drinking (filtered) water, and then was made available for consumption" where has this methodology been applied before? What is the treatment efficiency of this injection method? How was the exact injection of 30 mg/kg/day controlled, since there were 2-4 animals per box and how each one consumed the exact dose stipulated by the authors? Is this method better than the commonly used gavage administration?**

*We thank the reviewer’s questions. The method used in our study is a method commonly used in research studies involving the administration of medications to laboratory animals. Comparing this method to the gavage administration, each method has its advantages and limitations. The choice of administration method depends on various factors, including the characteristics of the drug, the study design, and the welfare of the animals. The priority for animal welfare in research has been steadily advancing and debatable. This alternative method to oral gavage was chosen based on the Three Rs principles in animal experimentation, specifically the ‘Refinement Principle’, which aimed to minimize or alleviate distress and improve the welfare of the animals during the study period. While gavage administration allows for precise control over the individual dosage and eliminates concerns related to group housing, it may cause some stress and discomfort to the animals. On the other hand, administering the medication via drinking water can be less invasive and may reduce the stress associated with handling and gavage.*

*Non-invasive drug administration methods are being considered by the scientific community. Similar approaches have been reported in various studies involving the administration of drugs via drinking water (such as presented in Kost Jr & Jackson, (1995) – DOI: 10.1161/01.hyp.25.1.82; Jaworska et al, (2017) – DOI: 10.1371/journal.pone.0189310; Naessens et al, (2023) – DOI: 10.1038/s41598-023-30515-0; Maida et al, (2016) –. DOI: 10.1097/HJH.0000000000001112; and others).*

*The exact dosage of 30 mg/kg/day was precisely controlled by adjusting the concentration of the dissolved medication in the water based on the daily water consumption of the rats. By monitoring and adjusting the concentration, the authors aimed to maintain a consistent dosage for each rat. Unfortunately, we were unable to accurately measure the individual consumption of each animal during the protocol, although we did measure water and feed consumption. Water intake was similar between the treated groups (OH and OTH), suggesting that the differences in arterial pressure control were indeed due to the addition of exercise. Additionally, similar arterial pressure reduction was observed throughout treatment between the HCTZ treated groups (OH, previously referred to as the OSH group, and OTH) compared to the non-treated groups (C, previously referred to as the S group, and OT), demonstrating the effectiveness of the treatment in maintaining arterial pressure at lower values than those observed in the hypertensive ovariectomized group (O, previously referred to as the OS group. Therefore, in addition to being easily replicable and in accordance with the increasingly emphasized principles of animal welfare in experimental research, our study adds further evidence that the method of drug administration by dilution in drinking water is effective and provides accurate information on the effects of consumption in rodent (rat) model studies.*

**Did the authors consider the half-life of the drug hydrochlorothiazide used in this study? The half-life of administration of a single dose of hydrochlorothiazide varies between 6 and 9 hours, with a peak effect between the first 4 and 6 hours of injection, with a maximum total duration of 12 hours, when considering interval doses, which are composed of two daily doses half-life ranges from 8 to 15 hours, with peak effect not determined, duration of effect 16 to 24 hours. But the animals ingested varying amounts of water with the compound during the day, how is the efficiency of this treatment ensured? It was described by the authors "The daily consumption was monitored and then considered to adjust the amount of water for the groups undergo to the drug treatment." Does this method ensure that all animals consume 30 mg/kg/day? How this was done, I need better explanations of this method.**

*We thank the reviewer for the important question. During all protocol and period of the hemodynamic assessments conducted, all* *animals were subjected to the same conditions regarding drug availability and duration of drug exposure. Except during the procedures (surgeries, training, AP recording, etc), hydrochlorothiazide was available 24 hours a day for all rats. In this regard, the onset time of the drug's action in the body was the same for all treated groups during the assessment. Moreover, each rat was placed in individual cages on the day of the catheterization procedure, during which the drug was provided individually to each rat at least 24 hours prior to the hemodynamic assessments of arterial pressure, baroreceptor sensitivity, and pressor system blockade. On the day of the hemodynamic assessments, the drug availability was maintained until the rats began the AP recording. After this evaluation, the drug was made available again to each rat, remaining accessible until the time of euthanasia, which was conducted in the next day.*

*Considering the reviewer comment, we added a sentence in the methods to clarify this important point: “All animals were allocated into individual cages and subjected the same conditions regarding hydrochlorothiazide availability and duration of hydrochlorothiazide exposure before and after hemodynamic assessments, remaining until euthanasia” in the Materials and Methods section (Hemodynamic and cardiac functional assessment).*

**Extensive revision would be necessary in view of the abbreviations used in the manuscript, as many of them are mentioned without using the full name, thus making reading unfeasible.**

*We thank for this comment. We have reviewed the manuscript and appropriately mentioned the full names and abbreviations.*

**In the topic " Inflammatory mediators", How was the tissue prepared for the analysis of inflammatory gauges, as the authors only describe that the heart was collected and that a commercially available ELISA kit (R&D Systems Inc.) was used to assess levels of TNF-alpha and interleukins 6 (IL-6) and 10 (IL-10) in cardiac tissue using microplate method. Was the tissue homogenized? Was it used in its entirety? How were these samples processed?**

*We thank the reviewer for these questions. We have added a detailed description of the analysis of inflammatory gauges in the Supporting Information Files.*

**These sentences "The recorded data were analyzed on a beat-to-beat basis to quantify changes in SAP, diastolic (DAP), mean AP (MAP), and HR (19,22).", due to not using correct abbreviations without prior description, it is not known the purpose of the technique or what data it provides.**

*We insert the correct abbreviations appropriately in the text.*

**There is a lack of a topic that addresses anthropometry used in this study, listing which organs were collected and processed, since in view of the results, the authors exchanged data on animal weights, orb weight, adipose tissue weight, and none of this is listed in the methodology of this study.**

*Thank you for important observation. We have added the proper description listing anthropometry and tissue collection in the Material and Methods section named ‘Anthropometry and tissue collection’.*

**Results and discussion**

**In all the topics present in the discussion, I advise the authors to start the paragraphs, explaining the importance of the analysis and the reason for it, so that they have a more fluid line of reasoning. As an example in the topic "Baroreflex sensitivity " the authors start the topic as follows: "Both trained groups (OT and OTH) presented an increased bradycardic response to phenylephrine compared with the OS group (OT: −1.3 ± 0.4 and OTH: −1.6 ± 0.3 vs. OS: −0.6± 0.3 bpm/mmHg) (p < 0.001)", the die is just thrown in the topic without a beginning of content, it would be more fluid if it started " before the analysis of the sensitivity of the baroreflex, we could notice that ..."**

*Thank you for suggestion. We have added a brief sentence explaining the importance of each analysis throughout the topics presented in the ‘Results section’. Changes were highlighted in the results section.*

**In front of the topic "Anthropometry", in this topic the authors present the results in a descriptive way, saying for example "However, weight gain during the study was higher in rats treated with HCTZ alone (OSH vs. S group)", but they do not present the values nor how much was the difference between the groups, I suggest identifying in percentage for example. The data in this topic are being sent as supplementary material, however I think it necessary to add the table as solid manuscript results, as they are discussed in the next topic. In the same table of the supplementary material for data on muscles such as soleus and Plantaris, which, when discussed in the topic, are described only as skeletal muscle, I suggest that they be listed, since each of these muscles has a different function and has different metabolisms, they are activated differently in each of the types of exercises performed in this present study.**

**This topic needs to be rewritten so that it can resemble the others, together with its data it should appear during the topic and not as supplementary material.**

*We agree with the reviewer suggestion. We added the respective values in percentage, as suggested.*

*We have moved the anthropometric data (S1 Table) from the ‘Supplementary materials’ to the results of the manuscript, as suggested by the reviewer. We changed “S1 Table” to “Table 1”.*

**In the topic "Tests of maximum exercise", the following sentence: "For the maximal running test, there were time (p < 0.001), group (p < 0.021) and interaction effects (p < 0.001). " Needs modifications, as cannot be understood, it was not possible to understand the intention and function of the sentence.**

**In this same topic, I suggest that the figure in front of these data are not explained through supplementary material, I believe that these data are of great value for the consolidation and understanding of the results exposed here, being part of the main data of your study.**
*We thank the reviewer for these comments. In the statistical analysis of repeated measures, which aims to compare the pre- and post-effects of a specific intervention at different time points throughout the protocol, it is important to consider the possibility of finding statistical differences related to the time of evaluation (differences between assessments), groups (differences between groups), and the combined effect of both factors, time and group (named ‘interaction effect’, indicating a distinct treatment effect over time). The commonly used writing style for this type of analysis involves describing the effects investigated in the statistical analysis. In this sense, we have revised and rewrote the sentence to improve the readability of the findings, as requested by the reviewer.*

*Additionally, we have moved the figures “S1A” and “S1B” from the ‘Supplementary materials’ to the results of the manuscript, as suggested by the reviewer. We changed figures “S1A” and “S1B” to “Fig 1A” and “Fig 1B”.*

**In the topic "Tail plethysmography", I once again reinforce the need to verify all the acronyms present in this article, so that each one of them is fully described.**

*We have reviewed the mentioned topic, as well as the article and made the necessary adjustments to describe the acronyms used in the study as requested by the reviewer.*

**In the discussion the authors say "However, we found that the non-exercised HCTZ-treated group gained more body weight and had more white adipose tissue weight (see S1 Table). In contrast, body weight gain and white adipose tissue were similar in control and trained groups, suggesting that the medication approach can induce anthropometric changes even in the absence of increased feed intake", how is it possible to explain weight gain in the non-exercised HCTZ-treated group, considering that food consumption did not show differences? organism, by which route would the amount of adipose tissue increase?**

*Thanks for your comment. We believe that this result is not solely explained by a single pathway. However, we believe that the participation of the renin-angiotensin system may be one of the possible mechanisms related to the anthropometric effect of hydrochlorothiazide in our study model. As mentioned in our discussion, hydrochlorothiazide has been linked to insulin resistance and fat storage (Eriksson et al, (2008) – DOI: 10.1161/HYPERTENSIONAHA.108.119404). According to this study, elevated levels of serum alanine aminotransferase, aspartate aminotransferase, and γ-glutamyl transferase after hydrochlorothiazide would explain the observed increase in hepatic adipose tissue. Fat deposition in the liver can occur when the capacity/function of adipose tissue storage is dysregulated.*

*The literature has shown an increase in the renin-angiotensin system after hydrochlorothiazide (Jessup et al (2008) – DOI: 10.1016/j.jash.2007.10.005). In this regard, the production of angiotensinogen by adipose tissue also promotes adipogenesis, and angiotensin II promotes the process of adipose tissue accumulation by increasing the production of key lipogenic enzymes and inducing lipogenesis through the AT2 receptor pathway (Pahlavani et al, (2017) – DOI: 10.1002/cphy.c160031). Conversely, a reduced accumulation of lipids and decreased expression of genes involved in adipogenesis, such as Ppar-g, Cebpb, Srebf1, and Adig, have been observed in murine adipocytes in vitro under conditions of angiotensinogen knockdown (Carroll et al, (2013) – DOI: 10.3389/fendo.2013.00010). On the other hand, exercise training has been positively related with down-regulation of vasoconstrictor axis of the renin-angiotensin system. Therefore,* *the lack of greater weight gain in the trained group treated with hydrochlorothiazide could be explained by the supposed positive modulation induced by exercise training on the renin-angiotensin system, as well as by the likely higher energy expenditure throughout the bouts of training.*

*Considering the reviewer comment, we added information regarding this important topic at the end of the 2^nd^ paragraph of the discussion.*

**The paragraphs "HCTZ has been linked to metabolic disorders, such as insulin resistance and fat storage (12). In our study, we did not ..." and "Performance in the treadmill exercise test improved in both trained groups, as previously reported (18), but sig ...", do not have a discussion, not the explanation of the mechanism, the authors only report again the data found. In this discussion topic it is necessary that the data be compared with others found in the literature.**

*Considering the questions raised in the previous item, we included in the manuscript (2^nd^ paragraph of the discussion) possible mechanisms that would explain the weight gain in the non-exercised group treated with hydrochlorothiazide, as well as the effects of exercise training in this condition.*

*However, the literature lacks evidence that experimentally evaluated exercise performance in exercise tests in female rats subjected to hydrochlorothiazide treatment, which limits us from making comparisons with our findings. On the other hand, some clinical studies have presented results of exercise tests in individuals treated only with hydrochlorothiazide or combined with another medication. Considering the reviewer comment, we included in 3^rd^ paragraph of the discussion two clinical studies that mention these effects of antihypertensive treatment.*

**Following the discussion, the authors say: "The initial dose chosen in monotheraphy to achieve adequate control of AP using HCTZ in hypertensive individuals is 25mg daily. Inadequate AP control after medication treatment, on the other hand, is common, and strategies such as increasing the dose and/or frequency, concurrent use of diuretics, or combination with other classes of antihypertensive are used." Who recommends these doses? Why is it necessary to change the dose? how does it affect increasing the daily dose or fractioning it for example? These responses are very important to support the choice of dose used in this article, what do the guidelines for the treatment of arterial hypertension say about this drug, what is the best dose, what frequency? are essential answers to be addressed in the present study and in the present discussion.**

*There is no precise recommendation regarding the optimal dose or frequency. These issues are individually adjusted in clinical practice, where the initial choice of medication should consider pharmacological aspects, the presence of associated diseases and end-organ damage, and the patient's socioeconomic conditions. Considering the important observations provided by reviewer, we have rewritten the sentence and added an actual Guideline of Hypertension (Barroso et al, (2020) – DOI: 10.36660/abc.20201238) to support our information.*

*It is important to mention that* *the dose of hydrochlorothiazide chosen in our study was selected with the aim of promoting arterial pressure reductions similar to the proposed exercise training (based on previous experiments where we observed a similar magnitude of arterial pressure reduction induced by exercise training in this model). Indeed, our arterial pressure results between the OH and OT groups demonstrated this similar effect. Thus, the pairing of the hypotensive effect between the approaches allows us to accurately assess which mechanisms (among those we evaluated) could be more benefited by medication, exercise training and/or the combination of approaches. Therefore, we added this information in the section “Pharmacological treatment”.*

**In this paragraph " The baroreceptor reflex regulates moment-to-moment the AP and plays a key role for maintaining cardiovascular homeostasis. Exercise training has previously been shown to improve baroreflex sensitivity in female ovariectomized rats (17,18,22). In the present study, we showed that ovariectomy reduced the tachycardic responses of baroreflex sensitivity. However, CET alone or combined with HCTZ improved both bradycardic and tachycardic responses of baroreflex sensitivity. The bradycardic response is more vagal dependent, while the tachycardic responses is more sympathetic-dependent. The increase and decrease of the vagal and sympathetic activity, respectively, has been cumulatively supported as a positive adaptation to exercise training. We hypothesize that improvements in vagal modulation, as measured by the increase in HF band and indirectly assessed by HRV, contributed to improvements in bradycardic responses. Although our study found no improvement in vagal modulation in the OT group as measured by HRV, it is possible that CET alone increased vagal tonus to the heart (32), as evidenced by the expressive resting bradycardia seen in the OT group. In the same way, it is possible that reduction in cardiac, vascular and overall sympathetic contribution, as verified by the reduction of the LF-PI (OTH group), LF-SAP (OT and OTH groups) and the lower reduction of the MAP after hexamethonium injection (OTH group), respectively, support the enhanced tachycardic responses". on the reflex baroreceptor? How does deprivation of hormones impact on the reflex baroreceptor? How does exercise along with pharmacological treatment impact on the reflex baroreceptor?**

*It is well established that ovarian hormones have considerable effects on the cardiovascular system. We have previously demonstrated that the reduction in estrogen concentration during the estrus phase may attenuate baroreflex sensitivity during the reproductive phase of life, suggesting that the hormonal oscillations of the menstrual cycle seem to directly affect this important cardiovascular reflex. (Ferreira et al, (2020) – DOI: 10.1186/s13293-020-00290-y). The injection of 17β-estradiol improved baroreflex-mediated control of renal and splanchnic nerve activity in ovariectomized rats, promoting an inhibitory action on the sympathetic nervous system (He et al, (1998) – DOI: 10.1152/ajpregu.1998.275.4.R1202.). In a similar manner, increased baroreflex control of the sympathetic nervous system resulting in subsequent decrease in arterial pressure has been observed following estrogen injection into the solitary tract nucleus, rostral ventrolateral medulla, and parabrachial nucleus in ovariectomized rats. (Saleh et al, (2000) – DOI: 10.1016/s0006-8993(00)02757-8). In young female, there is an increase of baroreflex sensitivity in phases of estrogen preponderance, whereas progesterone seemed to antagonize this effect (Minson et al, (2000) – DOI: 10.1161/01.cir.101.8.862).*

*The potential of exercise training in promote improvement in baroreflex sensitivity is well recognized. The improvement in baroreflex sensitivity after the combination of antihypertensive pharmacological treatment (enalapril) and exercise training has been observed by other authors (Maida et al, (2017) – DOI 10.1016/j.autneu.2016.12.002). In our study, we believe that the improvement observed in the group that combined the approaches was likely determined by the potential effect of exercise training, as the group treated only with hydrochlorothiazide did not show significant changes. An improvement in the baroreceptor sensitivity leads to an improvement in the autonomic discharge on the heart and vessels.*

*We revised the topic mentioned by the reviewer and improved the discussions (5^th^ paragraph).*

**In the following sentence "However, the influence of the HCTZ on sympathetic activity are inconsistent. HCTZ has been related to increased response to supine and upright MSNA (34)." What would MSNA be? Once again here I say, it would take an extensive review of the abbreviations. Also, does this sentence make no sense? Does it need to be rewritten?**

*We appreciate the comment and for emphasizing the need for abbreviation review. We agree that the mentioned sentences do not make sense. We have chosen to rephrase the paragraph to clarify (7^th^ paragraph)..*

**The following sentence "HTN is associated with end-organ damage. Although AP level management is the focus of treatment, its variability is another factor that requires attention due to its contribution to end-organ damage. High APV has been linked to decreased and increased cardiac (39) and renal function (39) and injury (40), as well as all-cause mortality (41)." This is displaced from the discussion, after introductory data, and does not enter the scope of the article when we stop to think about results related to this work.**

*We thank the reviewer for comment. We agree that the sentence is displaced from the discussion and partially excluded the highlighted section. However, we consider important to mention the scientifically demonstrated consequences of high arterial pressure variability in the context of arterial hypertension to reinforce the significance of evaluating this parameter in our study. Moreover, considering the previous reviewer comments regarding cardiac functional evaluation, we added the RPP in this version of the manuscript. We also showed measurements of inflammation, pro-oxidants, and oxidative damage in cardiac tissue, such as cytokines, NADPH oxidase, hydrogen peroxide, lipoperoxidation (TBARS) and protein oxidation (carbonyls). Together, we believe that these data support the hypothesis of cardiac end organ damage. Thus, considering the reviewer comment, we have reviewed and rewritten this section of the discussion (8^th^ paragraph).*

**The authors need to correct the references, as some are non-standard or follow different formats, for example in the sentence "We previously show that anti-inflammatory cytokines after ovariectomy, and that aerobic and resistance exercise training improved renal and that aerobic and resistance exercise training improved renal 18 and cardiac inflammation in ovariectomized SHR (18,20)."**
*We reviewed the formats of the references in the sentence and appropriately rewrote the sentence.*

**"Considering the anti-inflammatory effect of exercise in several diseases that has been widely described in the literature (44), and based on our data, it is plausible to speculate that exercise training contributed more to this adaptation in the OTH group than HCTZ", Which cytokines? what mechanisms? how does aerobic or anaerobic exercise modulate these anti-inflammatory pathways? How is this important for arterial hypertension?**

*Considering the reviewer comment, we have reviewed and rewrote the topic. Changes were highlighted in the discussion of the manuscript (9^th^ paragraph).*

**The authors wrote that "We found high pro-oxidant (NADPH oxidase) reduced antioxidant CAT enzyme activity and increased protein damage (carbonyls) in cardiac tissue of the OS group", however the phrase is meaningless, making it difficult to understand, I believe it is a problem in the translation from English, it would be necessary to correct the English in relation to this manuscript.**

*We agree that the sentence is incorrectly written. We reviewed the sentence highlighted by the reviewer and appropriately rewrote (11^th^ paragraph).*

**"Evidence evaluating the effect of HCTZ on oxidative stress showed inconsistent findings." Where was this published? Which authors?**

*We thank your comment. We review this paragraph to clarify, and references were added to each information. Changes were highlighted in this paragraph of the discussion (12^th^ paragraph).*

**"Importantly, CET combined with HCTZ reduced NADPH oxidase and hydrogen peroxide, enhanced CAT, SOD and GPx activities and decreased protein oxidation in cardiac tissue." What mechanism of activation of these enzymes in the face of the redox state induced by physical exercise?**

*It has been demonstrated a positive relationship between norepinephrine and activation of NADPH oxidase and superoxide anion, as well as TNF-α in the heart, resulting in a production of cytokines by the NF-κB. In this sense, exercise training could promote a reduction in norepinephrine levels, resulting in an inhibitory effect on pró-inflammatory and oxidative stress profile, reflecting in an improved cardiac function (Briones & Touyz, 2009 – DOI: 10.1161/HYPERTENSIONAHA.109.136622). Furthermore, nuclear factor erythroid 2-relatede factor 2 (Nrf2) is a most important transcription factor recognized for regulating antioxidant response, acting on a specific portion of DNA, known as AREs [antioxidant response elements], encoding antioxidant enzymes, such as SOD, CAT, and GPx, thus increasing the expression of antioxidant defense (Done & Traustadóttir, (2016) – DOI: 10.1016/j.redox.2016.10.003). Importantly, exercise training promote activation of the Nrf2 (Done & Traustadóttir, (2016) – DOI: 10.1016/j.redox.2016.10.003). We thank the reviewer question, and we added these mechanisms in the discussion (13 ^th^ paragraph).*

**"Inflammatory mediators are known to active pro-oxidants and influence redox balance. In this regard, the current study found that an increase in cardiac IL-10/TNF-α was associated with lower protein oxidation levels." Here the authors resumed saying the same thing as said above, about inflammation and the redox state, it would be better to unite the paragraphs and generate more links between these data.**

*We thank your suggestion. We reformulated the paragraphs related to the topics highlighted by the reviewer to improve the link between data and avoid redundant sentences (15^th^ paragraph).*

**"Antihypertensive medications have greater hypotensive effects at higher doses. Their side effects, however, are dose proportional. About this, tolerability may be compromised, and the individual may choose to discontinue treatment in the long run." Who described this previously? Where is it published? What do the guidelines say about?**

*We consider that the sentence is inaccurately written. We have rephrased the beginning of the sentence and included references that support the retained information (16^th^ paragraph).*

**Finally, I leave the following points as needs of this article, in addition to those already highlighted above:
1- correction of acronyms**

**2- English correction**

**3- Improvement of the discussion, as it seems like an instruction, as there is no discussion with data already published in the literature**

*Thank you for comments.*

*1 – We have reviewed the manuscript and appropriately corrected the use of acronyms throughout the manuscript*

*2 – We have submitted our manuscript to an English language grammatical and structure review.*

*3 – We changed the discussion as requested by both reviewers.*
