# Supplementary material for: Concurrent exercise training induces additional benefits to hydrochlorothiazide: Evidence for an improvement of autonomic control and oxidative stress in a model of hypertension and postmenopause
Source: PLoS One. 2023 Aug 7;18(8):e0289715. doi: 10.1371/journal.pone.0289715 (PMC10406179; doi:10.1371/journal.pone.0289715)
Supplement: Attachment — Submitted filename: Response to Reviewers.docx [file pone.0289715.s005.docx]

*Manuscript: PONE-D-23-10192R1*

*The authors would like to thank the Academic Editor and Reviewer for their important considerations. We considered the suggestions for the present study.* ***Responses are provided below, and changes made in the manuscript are highlighted****.*

**Academic Editor:**

**The new title is grammatically incorrect since "improve" is a verb. Suggestion: Concurrent exercise training induces additional benefits to hydrochlorothiazide: evidence for an improvement of autonomic control and oxidative stress in a model of
hypertension and postmenopause.**

*We appreciate the important observation of the Academic Editor. We rewrote the new title following the suggestion.*

**Reviewer #1:**

**I have no further comments. However, the authors may take more attention on limitations of their study and remind that IL6 is a myokine rather than a pro-inflammatory cytokine in the context of exercise.**

*We appreciate the valuable comments from the reviewer. We acknowledge the potential limitations as well as the strengths of our study. At this time, we will consider the comments to further enhance our results and interpretations.*
